# Supplementary material for: Household Income Is Related to Dietary Fiber Intake and Dietary Acid Load in People with Type 2 Diabetes: A Cross-Sectional Study
Source: Nutrients. 2022 Aug 7;14(15):3229. doi: 10.3390/nu14153229 (PMC9370737; doi:10.3390/nu14153229)
Supplement: Supplementary file 1 [file nutrients-14-03229-s001.zip › nutrients-1823742-supplementary.pdf]

Supplementary Materials

Table S1. Clinical characteristics of study participants according to included and excluded participants with type 2 diabetes mellitus.

	All<br>N = 314	Included<br>N = 201	Excluded<br>N = 113	*p*
Age (years)	69.0 (9.2)	68.8 (8.7)	69.3 (10.1)	0.708
Sex (men)	60.5 (190)	63.7 (128)	54.9 (62)	0.158
Duration of diabetes (years)	17.7 (11.2)	17.7 (10.9)	17.8 (11.7)	0.956
Family history of diabetes (+) (no data)	37.6 (118) (1.6 [5])	40.9 (81)	33.3 (74)	0.233
Height (cm)	161.5 (9.1)	162.2 (9.3)	160.4 (8.7)	0.105
Body weight (kg)	61.9 (11.7)	62.5 (11.8)	60.7 (11.3)	0.174
Body mass index (kg/m^2)	23.7 (3.8)	23.8 (3.5)	23.6 (4.1)	0.646
SBP (mmHg)	131.5 (17.7)	130.5 (16.3)	133.4 (19.9)	0.171
DBP (mmHg)	74.2 (11.9)	74.3 (11.5)	74.2 (12.6)	0.954
Antihypertensive drugs (+) (no data)	55.1 (173) (1.6 [5])	61.1 (121)	46.8 (52)	0.021
Presence of hypertension (+) (no data)	64.3 (202) (1.6 [5])	68.2 (135)	60.4 (67)	0.207
Insulin (+) (no data)	23.6 (74) (1.9 [6])	24.2 (48)	23.6 (26)	1.000
Smoking (+) (no data)	14.0 (44) (1.5 [5])	14.6 (29)	13.5 (15)	0.917
Habit of exercise (+)	51.0 (160)	57.7 (116)	38.9 (44)	0.002
Education level<br>(<12 years)	10.2 (32) (no data 21.3 [67])	13.0 (25)	13.0 (7)	1.000
Married status<br>(married/divorce/not married/bereavement)	60.2 (189)/<br>8.9 (28)/<br>6.7 (21)/<br>4.1 (13)<br>(20.1 [63])	77.3 (150)/<br>11.3 (22)/<br>6.7 (13)/<br>4.6 (9)	68.4 (39)/<br>10.5 (6)/<br>14.0 (8)/<br>7.0 (4)	0.284
HbA1c (mmol/mol)	54.9 (9.6)	55.8 (10.0)	53.3 (8.9)	0.032
HbA1c (%)	7.2 (0.9)	7.3 (0.9)	7.0 (0.8)	0.032
Plasma glucose (mmol/L)	8.0 (2.2)	8.0 (2.2)	8.2 (2.4)	0.412
Creatinine (umol/L)	78.4 (48.1)	75.3 (36.7)	84.0 (63.4)	0.128
eGFR (mL/min/1.73 m^2)	68.7 (21.4)	70.1 (21.3)	66.3 (21.5)	0.143
Renal failure (+) (no data)	5.4 (17) (2.2 [7])	5.1 (10)	6.4 (7)	0.832
Uric acid (umol/L)	308.7 (77.6)	310.5 (73.7)	305.6 (84.3)	0.599
Triglycerides (mmol/L)	1.5 (0.9)	1.5 (0.9)	1.5 (1.1)	0.994
HDL cholesterol (mmol/L)	1.6 (0.4)	1.5 (0.4)	1.6 (0.4)	0.305
Household income (high) (no data)	17.8 (56) (35.0 [110])	26.9 (54)	66.7 (2)	0.378
Total energy intake (kcal/day)	1752.1 (621.9)	1727.7 (509.5)	1795.5 (784.2)	0.355
Energy intake (kcal/IBW kg/day)	30.5 (10.9)	39.8 (8.7)	31.7 (13.9)	0.137
Total protein intake (g/day)	73.5 (30.3)	72.8 (27.6)	74.7 (34.5)	0.582
Protein intake (g/IBW kg/day)	1.3 (0.5)	1.3 (0.5)	1.3 (0.6)	0.321
Protein intake (% Energy)	16.8 (3.4)	16.8 (3.3)	16.9 (3.5)	0.845

Animal protein intake (g/day)	44.9 (24.0)	44.6 (22.3)	45.3 (26.8)	0.804
Animal protein intake (g/IBW kg/day)	0.8 (0.4)	0.8 (0.4)	0.8 (0.5)	0.555
Vegetable protein intake (g/day)	28.6 (9.8)	28.1 (8.7)	29.4 (11.7)	0.277
Vegetable protein intake (g/IBW kg/day)	0.5 (0.2)	0.5 (0.1)	0.5 (0.2)	0.093
Total fat intake (g/day)	56.4 (24.0)	55.7 (21.1)	57.6 (28.5)	0.508
Fat intake (g/IBW kg/day)	1.0 (0.4)	1.0 (0.4)	1.0 (0.5)	0.274
Fat intake (% Energy)	29.0 (6.3)	28.9 (6.5)	29.0 (5.9)	0.922
Total carbohydrate intake (g/day)	220.0 (82.2)	215.4 (68.0)	228.3 (102.5)	0.184
Carbohydrate intake (g/IBW kg/day)	3.8 (1.4)	3.7 (1.1)	4.0 (1.8)	0.062
Carbohydrate intake (% Energy)	50.6 (8.7)	50.4 (8.8)	51.1 (8.4)	0.509
Dietary fiber intake (g/day)	12.2 (5.1)	12.2 (5.0)	12.3 (5.5)	0.802
Carbohydrate/fiber ratio	19.5 (6.9)	19.4 (7.1)	19.6 (6.4)	0.807
Alcohol consumption (g/day)	7.3 (16.9)	7.8 (17.0)	6.4 (16.8)	0.489
PRAL (mEq/day)	7.1 (13.3)	6.2 (12.6)	8.8 (14.4)	0.098
NEAP (mEq/day)	49.8 (11.5)	49.0 (10.7)	51.3 (12.7)	0.082

Data was expressed as mean (standard deviation) or percentage (number). The difference between group was evaluated by Student's t-test or chi-square test. SBP, systolic blood pressure; DBP, diastolic blood pressure; eGFR, estimated glomerular filtration rate; HDL, high-density lipoprotein; IBW, ideal body weight; PRAL, potential renal acid load score; NEAP, net endogenous acid production score.
